# Linking private, for-profit providers to public sector services for HIV and tuberculosis co-infected patients: A systematic review

**DOI:** 10.1371/journal.pone.0194960

**Published:** 2018-04-10

**Authors:** Mollie Hudson, George W. Rutherford, Sheri Weiser, Elizabeth Fair

**Affiliations:** 1 Global Health Sciences, University of California, San Francisco, San Francisco, United States of America; 2 Department of Medicine, University of California, San Francisco, San Francisco, United States of America; NPMS-HHC CIC / LSH&TM, UNITED KINGDOM

## Abstract

**Background:**

Tuberculosis (TB) is the leading cause of infectious disease deaths worldwide and is the leading cause of death among people with HIV. The World Health Organization (WHO) has called for collaboration between public and private healthcare providers to maximize integration of TB/HIV services and minimize costs. We systematically reviewed published models of public-private sector diagnostic and referral services for TB/HIV co-infected patients.

**Methods:**

We searched PubMed, the Cochrane Central Register of Controlled Trials, Google Scholar, Science Direct, CINAHL and Web of Science. We included studies that discussed programs that linked private and public providers for TB/HIV concurrent diagnostic and referral services and used Review Manager (Version 5.3, 2015) for meta-analysis.

**Results:**

We found 1,218 unduplicated potentially relevant articles and abstracts; three met our eligibility criteria. All three described public-private TB/HIV diagnostic/referral services with varying degrees of integration. In Kenya private practitioners were able to test for both TB and HIV and offer state-subsidized TB medication, but they could not provide state-subsidized antiretroviral therapy (ART) to co-infected patients. In India private practitioners not contractually engaged with the public sector offered TB/HIV services inconsistently and on a subjective basis. Those partnered with the state, however, could test for both TB and HIV and offer state-subsidized medications. In Nigeria some private providers had access to both state-subsidized medications and diagnostic tests; others required patients to pay out-of-pocket for testing and/or treatment. In a meta-analysis of the two quantitative reports, TB patients who sought care in the public sector were almost twice as likely to have been tested for HIV than TB patients who sought care in the private sector (risk ratio [RR] 1.98, 95% confidence interval [CI] 1.88–2.08). However, HIV-infected TB patients who sought care in the public sector were marginally less likely to initiate ART than TB patients who sought care from private providers (RR 0.89, 95% CI 0.78–1.03).

**Conclusion:**

These three studies are examples of public-private TB/HIV service delivery and can potentially serve as models for integrated TB/HIV care systems. Successful public-private diagnostic and treatment services can both improve outcomes and decrease costs for patients co-infected with HIV and TB.

## Introduction

Twenty years ago, the World Health Organization (WHO) declared tuberculosis (TB) a global health emergency. Despite a 45% reduction in deaths since that time, there still were an estimated 9.6 million new cases and 1.7 million deaths due to TB in 2016 [1]. Although the widely available short-course drug regimens for drug-sensitive TB have a 90% cure rate, TB is the leading cause of death due to an infectious disease worldwide [1].

Immunocompromised individuals are particularly susceptible to developing active TB. Worldwide, 10% of the people who developed TB in 2016 were HIV-infected; the large majority of these was in Africa [1]. TB is the leading cause of death among people living with HIV and accounts for 33% of deaths among HIV-infected individuals [1]. The HIV epidemic has profoundly impeded TB control, both on a population and an individual level [2], and these two overlapping epidemics have been termed “the dual epidemic” [3]. In 2004, WHO called for integration of TB and HIV services to improve healthcare outcomes and harmonize care for these two infections [4].

In order to address effective integration of diagnostic and referral services, it is necessary to understand how patients access health services in low-and-middle income countries (LMIC). In this paper, we use the term “integration” to refer to systems of care for patients co-infected with HIV and TB that have collaborated with the goal of making access and service delivery easier for the individual patient. It is estimated that globally 60 to 80 percent of TB patients [5] and more than half of HIV-infected patients [2,6,7,8] seek care from the private sector [5], that is, providers “who work outside the direct control of the state” [9]. This definition is inclusive of both for profit and not-for-profit health care providers, who have varying degrees of training and ability to provide services and whose practice may or may not be standardized in accordance with government guidelines [9]. In 2013, the WHO Global TB Program has called for a collaboration of public and private providers (referred to as a public-private mix, or PPM) to maximize integration of TB/HIV services while minimizing costs [10]. Numerous studies have documented the benefits of integrating TB/HIV services [6,11], as well as the benefits of engaging all care providers for delivery of TB care [12]. These benefits include reduced costs to patients [13,14], improved access to treatment [7], increased case detection and better treatment outcomes [12].

However, considerable challenges remain in testing and bringing to scale effective PPM models for TB/HIV integrated services. Given WHO’s call to integrate TB and HIV services to improve patient outcomes and reduce costs to patients and engage all care providers for the delivery of TB/HIV services, we systematically reviewed the literature on how for-profit private providers link to public-sector diagnostic and referral services for TB/HIV co-infected patients.

## Methods

### Search strategy

We systematically reviewed literature from the following electronic databases: PubMed, the Cochrane Central Register of Controlled Trials, Google Scholar, Science Direct (via Elsevier), Cumulative Index of Nursing and Allied Health Literature (CINAHL), and Web of Science. We included studies that discussed programs that linked private providers to public-sector diagnostic and referral services for TB/HIV co-infection and had been published by July 2014. We reported our search results using the Preferred Reporting Items for Systematic Reviews and Meta-Analyses (PRISMA) flow diagram [15, 16]. We also completed the PRISMA checklist in accordance with our manuscript [17].

For grey literature, we searched both Google Scholar and conference abstracts from the annual meeting of the International Union Against Tuberculosis and Lung Disease [18].

We used two search strings:

i) "Tuberculosis" AND "HIV" AND "private" AND "public".
ii) "Tuberculosis" AND "HIV" AND "private”

After the initial search, we changed our search terms and included the second search string. We made this change because authors often did not refer to the public sector as “public,” but instead referred to the sector as “the state,” “government,” “national TB control program,” etc. We did not include the terms “partnership” and/or “mix” in our search terms as this terminology implied a formal partnership, and we did not want to limit our search by this criterion (authors may use different terminology (other than “partnership” or “mix”) to describe a relationship that is analogous to a formal partnership; we did not want these studies excluded from our search results).

### Types of studies

We included studies if they discussed public-private delivery of TB/HIV integrated services. We did not limit our search by study type; both quantitative (experimental and observational) and qualitative studies were included in our analysis.

#### Inclusion criteria

We included papers that discussed public-private diagnostic and referral services for TB/HIV co-infected patient in LMICs. Specifically, we only included studies that discussed private for-profit provider diagnostic and referral services for TB. Although the relationships among non-governmental organizations (NGO), private-not-for-profit providers, and the public sector are important to analyze, they were beyond the scope of this systematic review. We did not limit our search to a particular time period or by language. We screened grey literature, reports, conference abstracts and published full text articles.

#### Exclusion criteria

We excluded studies that discussed only the public sector or the private sector (rather than the public and private sectors in relation to each other), studies that compared the public and private sectors, and studies that discussed either TB or HIV programs (rather than TB/HIV integrated programs). Similarly, we excluded studies that described a collaboration between non-governmental organizations and not-for-profit clinical entities and the public sector. We chose to exclude these studies because we are interested in sustainable solutions that involve the government and local private system. In our experience, international NGOs often implement temporary care systems, which are ultimately abandoned when the project is completed.

#### Quality assessment

As described in Legido-Quigley, who used similar criteria to describe integrated services, we did not conduct a quality assessment to “ensure a comprehensive descriptive review” given that studies may lack outcome measures [11].

### Data extraction

One author screened all titles and abstracts (MH). Two authors reviewed the remaining articles after initial screening. Two authors extracted data from the eligible studies.

### Data analysis and synthesis

We evaluated models of public-private integration for co-current TB/HIV diagnostic and referral services using WHO guidelines [11,19]. These guidelines classify integrated models as follows:

i) Patients enter the health system through a TB clinic and are referred to HIV testing and care (“TB refers”), OR patients enter health systems through HIV services and are referred to a TB clinic for screening for active TB (“HIV refers”)
ii) TB clinics test for HIV on site and refer patients to a separate service for HIV treatment, OR TB screening is undertaken in the HIV clinic and patients are referred to TB services for treatment
iii) Treatment for HIV and TB is provided in the same health facility

We modified these definitions to accommodate public-private linkages in this framework:

i) Patients test at a private site for either TB or for HIV and then are referred to the public sector for either HIV or TB testing, whichever was not done at the private site (Tier 1)
ii) Patients test for both TB and HIV at a private clinic and are subsequently referred to the public sector for treatment for both diseases (Tier 2)
ii) Patients are tested and treated for both TB and HIV in a private facility, but the treatment is subsidized by government (Tier 3)

**Fig 1:**
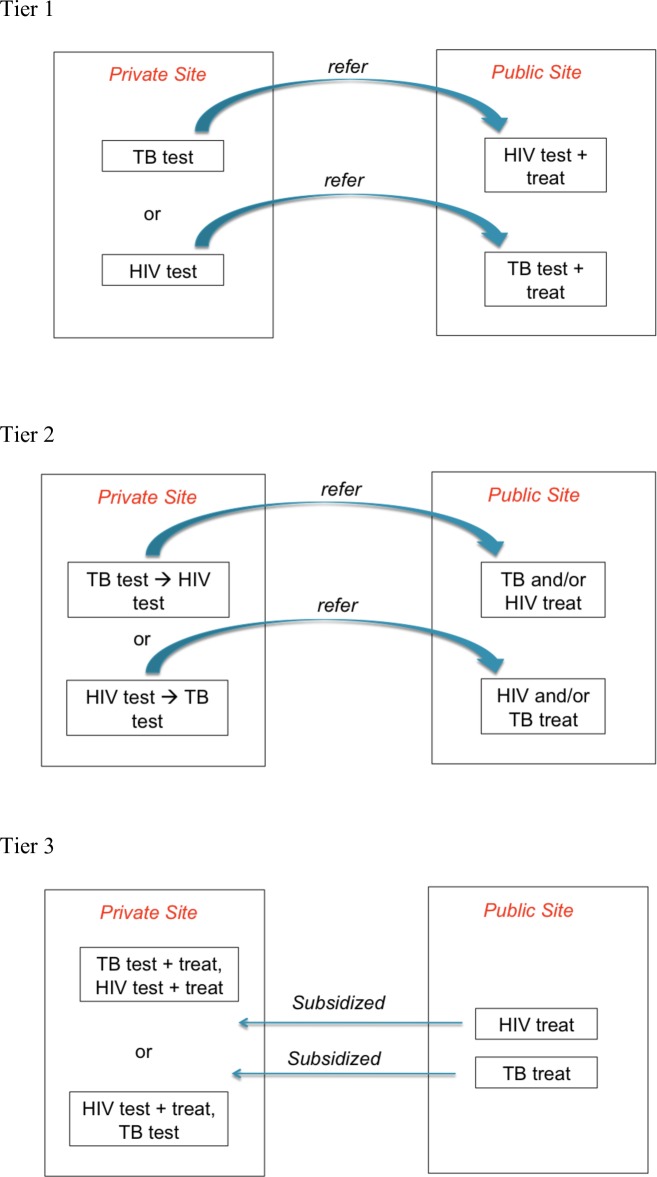
Relationships between private for-profit and public-sector providers. Tier 1 is the most basic model of public-private mixed service delivery. If patients tested positive for HIV or TB and can then be tested for TB or HIV at the same facility, then the health center is classified as a Tier 2 integrated health center. Health centers that can test for both TB and HIV and provide government subsidized medication for both conditions (effectively integrating public health service delivery in a private health setting) are classified as considered a Tier 3 integrated health centers.

Degrees of integration between public and private providers for TB/HIV diagnostic and referral services are illustrated in Fig 1. To classify health facilities described in eligible papers, we analyzed health centers according to two main criteria: 1) the ability of a private health facility to test for either TB and/or HIV and 2) the source of the medications private practitioners dispensed to co-infected patients. These criteria guided our categorization of health facilities by degree of integration of diagnostic and referral services. All eligible papers described situations in which patients first sought care in the private sector.

Because all eligible papers described a relationship between private health care providers and the public sector, all health centers met the criterion for Tier 1 integrated service delivery (i.e. the most basic model of public-private mixed service delivery). We then assessed each paper to determine if the health center(s) described offered services beyond the most basic of public-private mixed delivery of services (the most basic services being those described in Tier 1 of Fig 1). If a patient tested positive for HIV or TB and the same clinic could test for TB or HIV, then the health center qualified as at least a Tier 2 integrated health center. If the same health center were then unable to provide government subsidized medication for TB and/or HIV, then that health center would remain categorized as a Tier 2 integrated health center. However, if the health center could 1) test for both TB and HIV and 2) provide government subsidized medication for both conditions (effectively integrating public health service delivery in a private health setting) then the private clinic would be considered a Tier 3 integrated health center.

### Meta-analysis

Two authors independently extracted data from eligible papers [20,21] for two outcomes: 1) the number of TB patients who were tested for HIV, and 2) the number of patients initially diagnosed with TB who were given ART. We used Review Manager 5.3 (Cochrane Collaboration, Oxford UK) using a Mantel-Haenszel pooled risk ratio (RR) with a fixed effects model. We summarized dichotomous outcomes using a random effects model and calculating RRs and 95% confidence intervals (CI).

## Results

**Fig 2:**
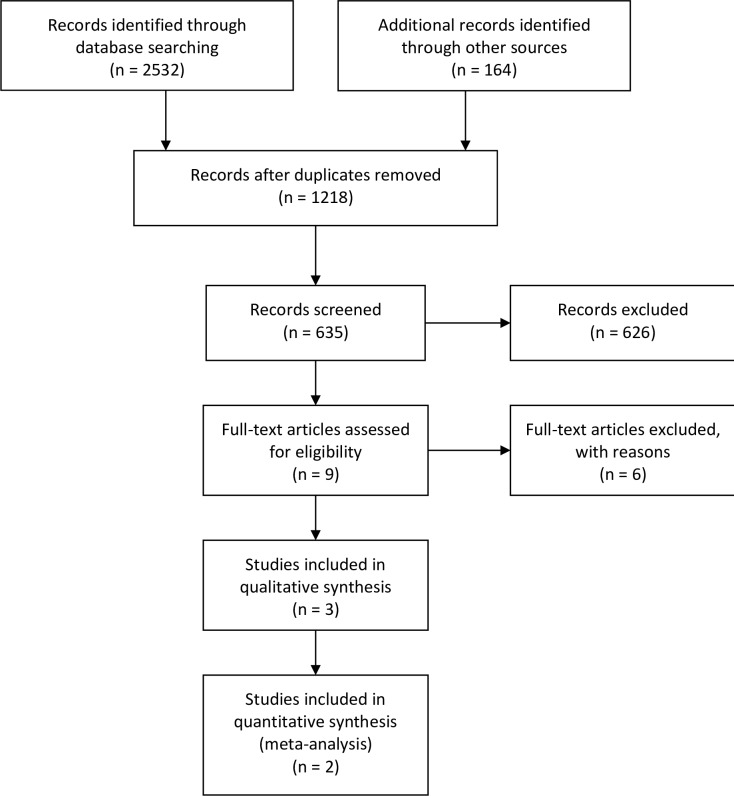
PRISMA flow chart of study selection. N = number of articles.

Our searched yielded 2,532 potentially relevant articles and 164 potentially relevant conference abstracts. After excluding duplicates, we screened 637 records. Of these, we were unable to recover two articles; an additional 626 articles did not meet our inclusion criteria on abstract and title review, and were subsequently removed. We assessed nine full text articles and included three in our final analysis [20–28] [Fig 2]. Six of the nine articles were excluded for our final analysis because they 1) described diagnostic and treatment practices in either the public or private sector, but not did actually describe the relationship between the private and public sectors; 2) described an intervention, rather than a discussion/analysis of an existing health system (the rationale for excluding these study types is described under our exclusion criteria); or 3) described treatment practices for both TB- and HIV-infected patients, but not for patients co-infected with both TB and HIV.

All three included studies were conducted in high-burden TB and HIV LMICs (India, Kenya, Nigeria). Daniel and colleagues evaluated the involvement of 20 private-for-profit clinics in TB and TB/HIV case finding in a state in Nigeria [20]. Outcomes were the proportion of TB patients who were tested for HIV, the percentage of TB patients who found to be HIV infected, and the percentage of TB and HIV-infected patients started on treatment for either TB or HIV. This study did not specify whether the clinics were located in rural or urban settings, but, as it was conducted in Lagos State, clinics were most likely urban or peri-urban. In Nigeria, the national TB program supplies TB medication to the private healthcare providers; those attending private practitioners only pay consulting fees. According to Ministry of Health guidelines, the national TB control program can also provide private practitioners with HIV diagnostic reagents. However, while some private providers did receive HIV tests from the state, other private providers did not. In other words, patients attending some private healthcare providers did not have to pay for an HIV test, while those attending other private providers were required to pay. The authors found that of 7,557 patients attending public clinics, 6,517 (86.2%) were tested for HIV while only 154 of 290 (53.1%) of those attending private not-for-profit clinics were tested (RR 1.62, 95% CI 1.46–1.81). However, among those found to be infected with HIV, those in enrolled in public clinics were no more or less likely to be started on antiretroviral therapy than those in private clinics (23.8% vs. 8.3%, RR 2.85, 95% CI 0.44–18.68). Given the descriptions of healthcare financing between the public sector and private providers, some facilities in this study qualify as Tier 3 integrated facilities (since patients were able to be tested and treated for both conditions in the same clinic, and received state subsidized TB and HIV medication), and other facilities fall between Tier 2 and Tier 3 in their degree of integration of diagnostic and referral services (because while TB medication was subsidized, HIV medication was not). The authors also found that HIV testing rates were lower among patients attending private for-profit clinics and speculated that the cost of an HIV test (priced between $5-$10 USD at the time of the study) may have partially accounted for lower testing rates.

Miller and colleagues qualitatively assessed HIV testing and referral practices among a purposeful sample of 15 private practitioners treating TB patients in five health centers of various types (i.e. government, private, and a combination of the two sectors) in Chennai, India [22]. These clinics that had integrated TB/HIV care, albeit with varying degrees of integration; the authors essentially investigated various delivery models for public-private TB/HIV diagnostic and referral services. The authors found that a few private health centers did not offer HIV testing or referral for TB patients. Although Indian national guidelines require private practitioners to refer patients who test positive for HIV to the public sector, private practitioners rarely referred their patients for additional testing and treatment. Private practitioners often do not feel obligated to practice according to national guidelines. Instead, they described using “‘expertise’ and ‘judgment’ to decide if a patient is “at risk” [29]. Such judgments are based on factors such as presence of clinical symptoms, behavioral risk factors, and the patient being of reproductive age. Additionally, private practitioners were sometimes reluctant to refer patients to the government hospital because they perceived public facilities to be of lower quality. Public practitioners interviewed in the study also reported feeling overwhelmed by their current patient load and felt they could not accommodate referrals from the private sector. Lastly, private practitioners were concerned that patients would be offended if they were offered an HIV test. In sum, these health centers would not meet any of the criteria for integrated services (i.e. they do not meet the basic requirements for a level one integrated system).

Practitioners who worked in both the private and public sectors reported different diagnostic and referral practices depending on in which sector they were working. For example, although a practitioner may not offer an HIV test in a private practice setting for fear of offending the patient, the same practitioner would offer the test while working in a public-sector hospital. At least one private practitioner in the study referred co-infected patients to the government facilities for HIV treatment. Such care would be categorized as Tier 2 service integrated testing and referral services (the private practitioner can test for both conditions and refers the patient to the public sector for subsided treatment). One private health facility met the criterion for Tier 3 integrated service and was the only facility that was considered to have an official public-private partnership in place. Private practitioners were able to test for both conditions, and they were able to treat patients with government-subsidized medication. The study reported that enforced guidelines in concert with an official partnership with the public sector allowed this care delivery model to function. Lastly, authors discussed how “linking” a patient to care (i.e., for treatment initiation) did not guarantee patient care beyond the first visit. Many patients lived far away from the health center and were unable to return for follow-up appointments and medications.

Chakaya and colleagues studied engaging private health care providers to provide TB care according to national guidelines and to report cases to the state in Nairobi, Kenya [21]. The authors described several outcomes, such as percentage of patients diagnosed in the private sector that were registered with the national program, the percentage of patients tested for HIV (across both sectors), and percentage of patients who began treatment for both TB and HIV. As of 2005, private providers in Kenya began to receive supplies of both ART and TB medications from the public sector. In this study, private providers in Nairobi were able to test for both TB and HIV and were supplied TB medications by the national program. However, the private practitioners described in the study did not have access to government-subsidized ART (despite a 2005 policy change). Of the 1,740 TB patients reported from private clinics in the study, 732 (42.1%) had been tested for HIV (with no reported comparison for public clinic patients). Among the 372 (51%) found to be HIV-infected, those diagnosed in the public sector were marginally less likely to be started on ART than those diagnosed in the private sector (32.0% vs. 42.1%, RR 0.88, 95% CI 0.77–1.01). Given that private providers can supply state-subsidized TB medication, but not ART to co-infected patients, private healthcare provision for co-infected patients would be considered between Tiers 2 and 3.

In a meta-analysis of the two quantitative reports, TB patients who sought care in the public sector were almost twice as likely to have been tested for HIV than TB patients who sought care in the private sector (RR 1.98, 95% CI 1.88–2.08) (Table 1). However, at the same time HIV-infected TB patients who sought care in the public sector were marginally less likely to initiate antiretroviral therapy than TB patients who sought care from private providers (RR 0.89, 95% CI 0.78–1.03) (Table 2). These data are consistent with findings from the one qualitative study we reviewed as well [22].

**Table 1:** Tuberculosis patients tested for HIV by sector.

Study	Public (tested/total)	Private (tested/total)	Risk ratio (95% CI)
Chakaya 2008	Not reported	732/1740 (42.1%)	Not estimable
Daniel 2013*	6517/7557 (86.2)	154/290 (53.1%)	1.62 (1.46–1.81)
Total	6517/7557 (86.2)	886/2030 (43.6%)	1.98 (1.88–2.08)

CI, confidence interval

*Private for-profit clinics only; private not-for-profit clinics excluded

**Table 2:** HIV-TB co-infected patients prescribed antiretroviral therapy by sector.

Study	Public (prescribed ART/total)	Private (prescribed ART/total)	Risk ratio (95% CI)
Chakaya 2008	2428/7589 (32.0%)	136/372 (42.1%)	0.88 (0.77, 1.01)
Daniel 2013*	369/1551 (23.8%)	1/12 (8.3%)	2.85 (0.44, 18.68)
Total	2797/9140 (30.6%)	137/384 (35.7%)	0.89 (0.78–1.03)

ART, antiretroviral therapy; CI, confidence interval

*Private for-profit clinics only; private not-for-profit clinics excluded

## Discussion

We identified three studies that met our eligibility criteria, representing different geographic regions and models of care. In reviewing both the quantitative and qualitative studies, we identified key success strategies and challenges to public-private integrated diagnostics and referrals. First, all authors described how supplying private providers with state-subsidized medication promoted initiation of treatment for both TB and HIV. Because patients who initially sought care from a private provider did not have to go to the public sector for medication, patients experienced fewer treatment delays. Moreover, although eligible studies were geographically diverse, parallel challenges emerged in all three. All discussed problems regulating the private sector, specifically assuring adherence to national guidelines. Regulation would help ensure that private providers followed standard testing and treatment procedures and did not charge patients for state subsidized medication. A lack of coordination, both within and across sectors (i.e., between vertical programs and provider types), also was identified as a challenge. Adequate coordination and communication are essential to a successful integration of diagnostic and referral services across sectors.

Although the number of articles we identified was small, our findings parallel those of other authors who have investigated the use of PPMs for other health services (in particular for non-HIV-associated TB). Additionally, the results of the qualitative study [22] supported our quantitative analysis. Several, for example, have concluded that supplying private providers with government subsidized medications, aligning incentives between private providers and the national program, orienting private providers to national and international guidelines [27], and establishing a better referral system [30, 31] are all key components of an effective PPM for TB control. Including these programmatic components can improve equity of access to higher quality care [27]. Incorporating such essential components as central to policy formation may prove to be highly beneficial and should be considered when developing PPMs for TB/HIV co-infected patients.

Our study has significant limitations. First, only three articles met out inclusion criteria. Such a small sample size makes meaningful analysis difficult. It should be noted that using search terms such as “private” or “public” may exclude articles that used different terms for the private and public sectors. Moreover, we would assume PPMs in different environments (i.e. rural versus urban) would require different interventions, but our study size was too small to adequately assess and categorize environment-dependent challenges. Lastly, the Centers for Disease Control and Prevention estimates that there are approximately four million “missing” cases of tuberculosis (in other words, patients who are infected with tuberculosis but whose infection is not recorded in tuberculosis databases) [32]. Because we do not know if, and/or where, these patients are receiving care, it is possible that these cases would affect our analysis. However, our findings closely paralleled those of other studies of public-private partnerships and integrated care service delivery for TB [30,31], which suggests that our results are at least reasonably robust.

Additional research is needed to understand the amount of care delivered by the private sector, the various types of private providers, additional models of public-private diagnostic and referral services, and why existing public-private services do not test and treat all patients according to protocol. Such research will require both quantitative and qualitative analysis and should be conducted in a range of environments (e.g., rural and urban). Developing strong PPMs for TB/HIV integrated service delivery will require strong national and international commitment from both private and public partners. These efforts, however, are worthwhile and will significantly contribute to the fight against the dual burden of HIV and TB.

## Supporting information

S1 Fig PRISMA checklist (page 1). (TIF)

S2 Fig PRISMA checklist (page 2). (TIF)
