# Supplementary figures and images for: Linking private, for-profit providers to public sector services for HIV and tuberculosis co-infected patients: A systematic review
Source: PLoS One. 2018 Apr 10;13(4):e0194960. doi: 10.1371/journal.pone.0194960 (PMC5892869; doi:10.1371/journal.pone.0194960)

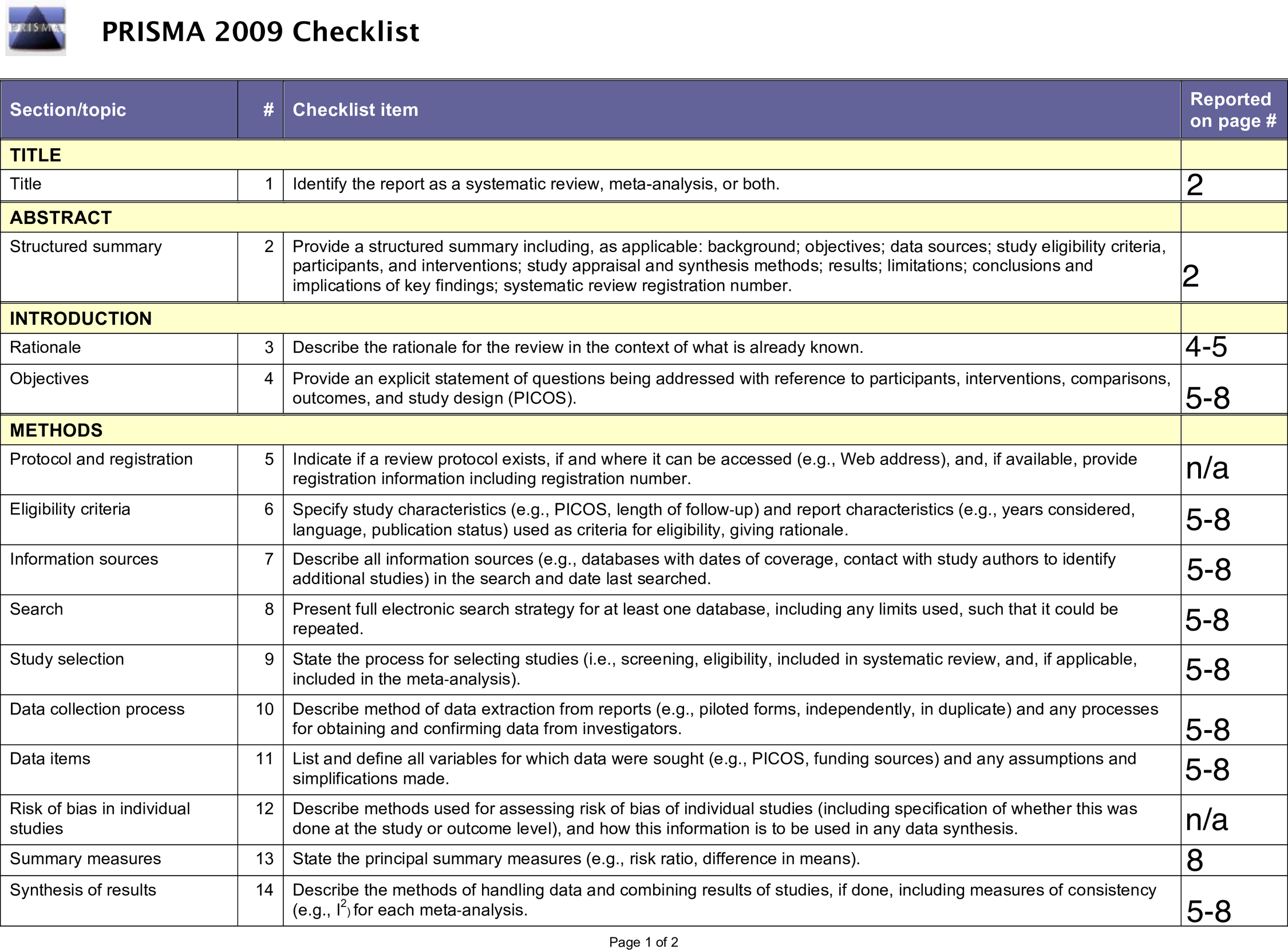

Supplement: S1 Fig — (TIF) [file pone.0194960.s001.tif]

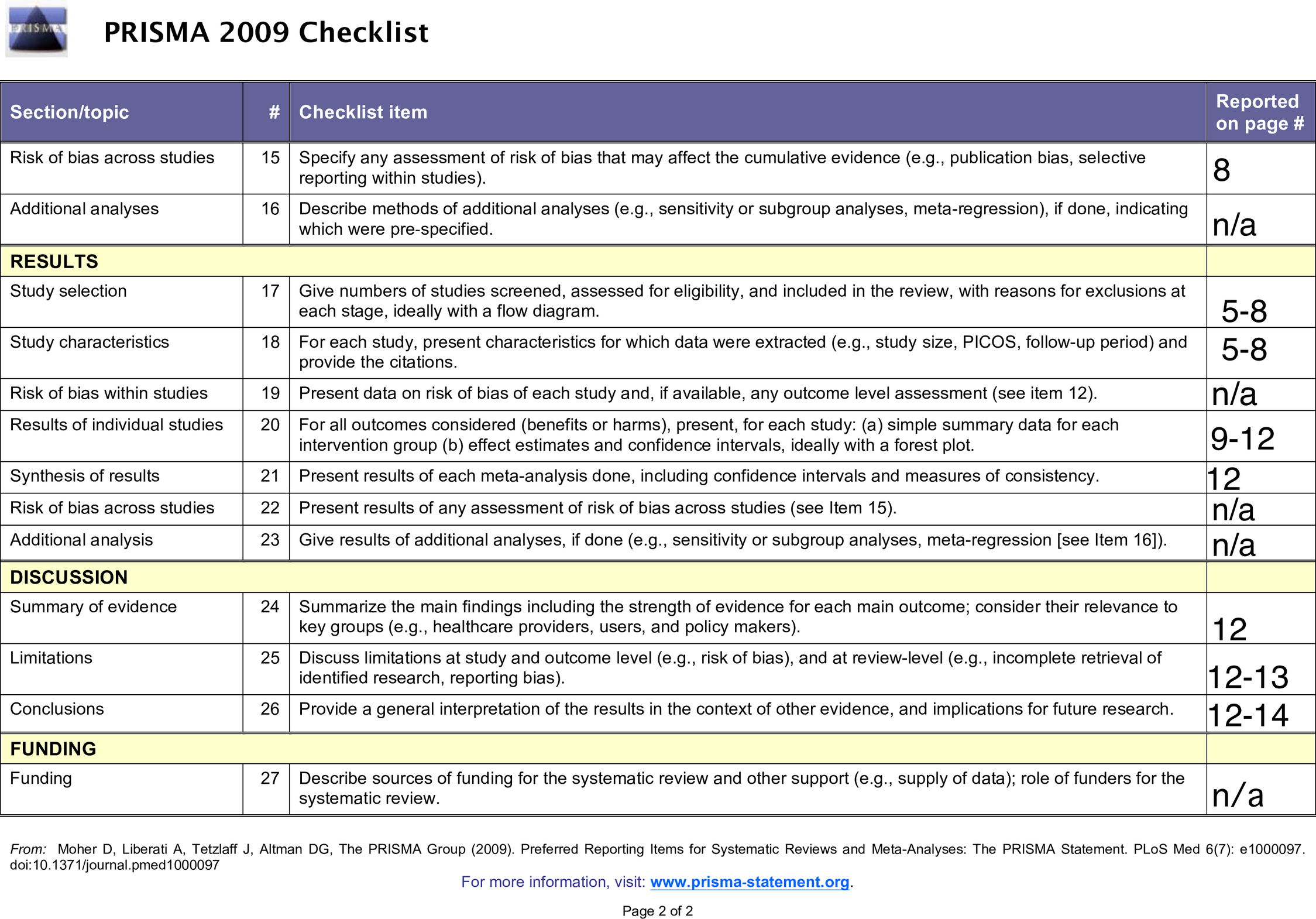

Supplement: S2 Fig — (TIF) [file pone.0194960.s002.tif]
